# Quality assessment of structure and language elements of written responses given by seven Scandinavian drug information centres

**DOI:** 10.1007/s00228-017-2209-3

**Published:** 2017-02-05

**Authors:** Linda Amundstuen Reppe, Olav Spigset, Jens Peter Kampmann, Per Damkier, Hanne Rolighed Christensen, Ylva Böttiger, Jan Schjøtt

**Affiliations:** 1 Faculty of Nursing and Health Sciences, Nord University, Namsos, Norway; 2 Department of Laboratory Medicine, Children’s and Women’s Health, Norwegian University of Science and Technology, Trondheim, Norway; 3 Regional Medicines Information and Pharmacovigilance Centre (RELIS Midt-Norge), Department of Clinical Pharmacology, St. Olavs Hospital, Trondheim, Norway; 4 Department of Clinical Pharmacology, Bispebjerg University Hospital, Copenhagen, Denmark; 5 Department of Clinical Biochemistry and Pharmacology, Odense University Hospital, Odense, Denmark; 6 Clinical Pharmacology, Department of Drug Research, Linköping University, Linköping, Sweden; 7 Section of Clinical Pharmacology, Laboratory of Clinical Biochemistry, Haukeland University Hospital, Bergen, Norway; 8 Department of Clinical Science, Faculty of Medicine and Dentistry, University of Bergen, Bergen, Norway; 9 Regional Medicines Information and Pharmacovigilance Centre (RELIS Vest), Haukeland University Hospital, Bergen, Norway

**Keywords:** Quality assurance, Health care, Drug information services, Information literacy

## Abstract

**Purpose:**

The aim of this study was to identify structure and language elements affecting the quality of responses from Scandinavian drug information centres (DICs).

**Methods:**

Six different fictitious drug-related queries were sent to each of seven Scandinavian DICs. The centres were blinded for which queries were part of the study. The responses were assessed qualitatively by six clinical pharmacologists (internal experts) and six general practitioners (GPs, external experts). In addition, linguistic aspects of the responses were evaluated by a plain language expert.

**Results:**

The quality of responses was generally judged as satisfactory to good. Presenting specific advice and conclusions were considered to improve the quality of the responses. However, small nuances in language formulations could affect the individual judgments of the experts, e.g. on whether or not advice was given. Some experts preferred the use of primary sources to the use of secondary and tertiary sources. Both internal and external experts criticised the use of abbreviations, professional terminology and study findings that was left unexplained. The plain language expert emphasised the importance of defining and explaining pharmacological terms to ensure that enquirers understand the response as intended. In addition, more use of active voice and less compressed text structure would be desirable.

**Conclusions:**

This evaluation of responses to DIC queries may give some indications on how to improve written responses on drug-related queries with respect to language and text structure. Giving specific advice and precise conclusions and avoiding too compressed language and non-standard abbreviations may aid to reach this goal.

**Electronic supplementary material:**

The online version of this article (doi:10.1007/s00228-017-2209-3) contains supplementary material, which is available to authorized users.

## Introduction

To promote rational use of medicines, drug information centres (DICs) give advice on therapeutic drugs in response to queries by health care professionals. The International Pharmaceutical Federation (FIP) has recommended quality assurance procedures like regular review of responses and periodic review of resources and procedures for DICs. However, easily applicable criteria for assessment of the quality of responses are not given [1]. No generally accepted quality criteria, nor guidance how to investigate these issues, exist for DICs [2].

The Scandinavian DICs are regional centres affiliated with units of clinical pharmacology at university hospitals. In Norway, the centres receive funding from the Ministry of Health and Care Services. In Sweden, centres are funded by the county councils, and in Denmark, the individual university hospital departments fund the centres. The centres produce written responses to drug-related queries from health care professionals [3–5]. To be able to use a response from a DIC in patient treatment, it is essential that the enquirer has the ability to read and understand both the language, content and specific data (e.g. numeric study results) presented in the response, i.e. has knowledge in drug information literacy [6] and numeracy [7], respectively. These issues have been extensively studied in the context of consumer information but may also apply for information given to health care professionals [8]. With respect to health care professionals, the practical usefulness of the responses is important. As an example, a qualitative study of the German Summary of Product Characteristics (SPCs) concluded that physicians found them disorganised and difficult to use [9].

In the last few years, there has been an increasing focus on the use of *plain language* in the information distributed by public agencies [10–12], including health systems [13]. *Plain language* may be defined as *the writing and setting out of essential information in a way that gives a cooperative, motivated person a good chance of understanding it at first reading, and in the same sense that the writer meant it to be understood* [14]. In Sweden, the use of plain language is actually regulated by law [15]. In Norway, a project called *Clear language in Norway’s Civil Service* was started in 2008 to stimulate public agencies to adopt good, user-friendly language [11]. Also, the Danish language council is committed to this goal [16].

The principles for plain language are based on scientific research; however, solutions how to achieve this goal are not clear-cut [17]. Positive results have been reported from the Norwegian *Clear language in Norway’s Civil Service* project [18], although it is difficult to attribute the results to the specific project as such. Although DICs primarily serve health care professionals, we do not know whether the use of customized plain language may be preferable. Even though health care professionals are familiar with medical terms, we cannot automatically assume that they understand all information in the responses. The text may be read in another context than it was produced, and this may affect how the message is interpreted [19]. Furthermore, some DICs, e.g. the Norwegian centres, publish responses in generally accessible databases and the users of these databases may have various knowledge related to the topic. General understandability of the text is therefore crucial.

The aim of this study was to assess the quality of written responses from Scandinavian DICs to drug-related queries by focusing on the qualitative aspects of the texts. Specifically, we aimed to identify elements increasing or decreasing the quality of written DIC responses, with emphasis on structure and language elements.

## Methods

Eleven Scandinavian DICs were presented with the protocol for the study, and seven chose to participate. Staff members at the centres were pharmacists and residents and consultants in clinical pharmacology. Key information of the centres is given in Table S1. We recruited 22 (three to four per DIC) general practitioners (GPs) familiar with each of the DICs to simultaneously send six identical drug-related queries by e-mail to each of the seven participating DICs. The queries (translated to English) are presented in Table S2. Authors LAR, OS and JS developed the queries in Norwegian. YB translated them to Swedish, and HRC to Danish. Some adjustments between countries were necessary (Table S2). The study period lasted 8 weeks from January to March 2013. The queries chosen were typical for those usually received by the centres; they had no clear-cut answers and concerned the most common query categories, including adverse effects, drug use in pregnancy and lactation, drug interactions and choice of drug therapy. Five queries were patient-specific and one was a more general query (Table S2). The study was reported to the Norwegian Social Science Data Services in accordance with national legislation.

Staff members at the participating DICs were informed about the study but were blinded in terms of which queries were study queries and which were not. The 42 responses given, seven to each of the six queries, were anonymised for which centre and staff member that had processed them. We chose seven clinical pharmacologists (six of the authors: two Norwegian, one Swedish and three Danish, and a second Swedish clinical pharmacologist) within the DICs (internal experts) to review the professional quality of responses. To maintain blinding, none of the authors were involved in preparing any of the responses to the study queries, or allocate queries to specific staff members during the study period. In addition, two Norwegian, two Swedish and two Danish GPs (external experts) familiar with the DICs were chosen to review the responses, as GPs are the most common group of enquirers. Participating GPs each received NOK 10,000 for their assessment. Their familiarity with the DICs would reduce the risk of misunderstandings related to the DICs’ role and function. Finally, we chose to include a Norwegian external plain language expert with a Master of Arts in Rhetoric, to review the language quality of the written responses.

The internal and external experts individually reviewed all 42 responses and assessed the quality of them using a form allowing quantitative scorings as well as qualitative comments. The presentation of quantitative quality assessments by internal and external medical experts in relation to time consumption when processing the responses has been presented in a separate article [20]. In the current article, we performed a content analysis of and present examples of qualitative comments made by the internal and external experts concerning the strengths and weaknesses of the responses. All experts were asked one specific overall question in the assessment form, requiring them to answer with a qualitative comment: *Describe your assessment of concordance in practical advice and conclusions between responses from the various centres to the same query. Specifically: Did the documentation found and/or advice given differ between responses?* In addition, qualitative comments of any kind were encouraged for each of the individual 42 responses. The degree to which individual experts commented varied largely, but 10 (six internal and four external) of the 12 experts commented regularly on many aspects of the responses.

### Analysis of qualitative statements

The author LAR manually reviewed and categorized comments into three main groups reflecting central themes in the comments: (i) presentation of background documentation and references/enclosed literature, (ii) provision of advice/conclusions and (iii) the use of definitions of concepts/explanation of abbreviations. Another author (JS) reviewed the categorization of comments. The condensation and selection of data and the selection of quotes presented was based on agreement between LAR and JS. A third author (OS) was consulted in case of controversy.

### Assessment of language quality

To evaluate the language quality of the responses, we contacted a company specializing in plain Scandinavian languages and involved in *Clear language in Norway’s Civil Service* [11]. The recruited Norwegian plain language expert had experience in evaluation of texts in all Scandinavian languages. A pilot study was performed, using six drug-related queries and responses taken from the Norwegian, Swedish and Danish DIC databases. The aim of the pilot study was to formulate relevant language quality criteria to assess the DICs’ responses, account for important professional and methodological caveats of the study and test the quality criteria on the basis of the findings. Eight quality criteria were compiled (Table 1). The plain language expert explained the background for each of them and used the criteria to assess the quality of the 42 responses. The expert evaluated responses written in Norwegian, Swedish and Danish. As one of the DICs presented their main responses in English, the preliminary responses (written in Danish) were assessed for this centre. The eight language criteria could be scored from 0 (poorest quality) to 4 (highest quality), and these scores were added giving a total score between 0 (lowest quality) and 32 (highest quality) per response.

**Table 1:** Criteria used to describe the quality of structure and language elements of written responses from Scandinavian drug information centres (DICs) to six fictitious queries (six queries were sent to each of seven different DICs)

Criteria no.	Criteria	Mean score (min to max)	Median score (0–4)	Comment
1	Does the response have a distinct structure?	3.1 (2 to 4)	3	The purpose of the structure is to help the reader get an overview, sort information and point out the more important parts of the response. The use of e.g. captions and paragraphs will help the reader to sort out information.
2	Are words and concepts used in the query, also repeated in the response?	2.5 (1 to 4)	3	The use of similar words and concepts in the response, as in the query, will lead to continuity and consistency. The repetition and explanation will increase the likelihood that the writer and the reader understand each other.
3	Are words and concepts explained or defined? Are other words used to explain them?	2.5 (1 to 4)	3	
4	Is the response written in an abstract or concrete style?	2.4 (1 to 3)	2.5	Very compressed texts may be more difficult to read and understand, and the use of words to make the text less compressed is preferred. One fact should be presented at a time. The use of active (“we assume this is caused by”) instead of passive (“it is assumed that this is caused by”) formulations is also preferred. This does not mean one have to be personal in his style, but a direct tone will make it easier for the reader to know exactly what action he/she should take.
5	Does the use of pronouns, verbs and dependent clauses help making the text less compressed?	2.5 (0 to 4)	3	
6	Is it easy for the reader to understand who should perform the described actions?	2.4 (0 to 3)	2	
7	Are answers to the query given scattered or as one common conclusion?	2.8 (1 to 4)	3	Is the query interpreted correctly, and all parts of the query responded to? It is crucial that the answer to the query is given in a conclusion. This is particularly important if the staff member has discussed several possible solutions and advice along the response. A conclusion in the end of a response has the benefits of summarizing the knowledge, and possibly, clarifying potential misunderstandings.
8	Is it easy to find a concrete answer to the query?	2.9 (1 to 4)	3	

The quality criteria were developed by a plain language expert with a Master of Arts in Rhetoric and are based on plain language theory [14]. Each criterion was scored from 0 (poorest quality) to 4 (highest quality)

## Results

The material assessed in this study consisted of 42 responses to the six queries. Extra documents were enclosed in 12 responses. Of these attached documents (*n* = 21), nine were former responses given by the DICs, seven were review articles, two were treatment recommendations, two were information obtained from drug information databases and one was an original article. The external and internal experts assessed all documents, whereas the plain language expert mainly assessed the written responses specific to the queries. We did not identify differences between the external and internal experts concerning the central themes and the assessment of responses; therefore, the results from the medical experts’ evaluations are presented together.

### Assessment by medical experts

A total of 334 comments were given. Table 2 presents examples of specific comments given by the internal and external experts to characterize elements of the responses affecting their quality. Table 3 shows the results of our analyses of characteristics that seem to increase or decrease the assessed quality of the responses. Examples illustrating the three main themes are given below.

**Table 2:** Examples of comments given by internal (clinical pharmacologists) and external (general practitioners) experts to assess the quality of 42 responses to drug-related queries (six fictitious queries posed to seven Scandinavian drug information centres (DICs)). Co﻿mments relating to all responses are named *general comments*. Comments relating to one specific response are named *specific comments*.

	Comments related to aspects increasing the quality of responses	Comments related to aspects decreasing the quality of responses
Internal experts	• Enclosed articles should be “to the point”, highly relevant and not too demanding for the enquirer to read (expert E, general comment). • Systematic and well-documented response that results in specific advice (expert G, specific﻿ comment). • Very thorough and deepened response with a review of primary literature. It depends on your preferences whether this is necessary, but the conclusion is precise (expert G, specific comment). • The best responses give specific and practically oriented answers, while the poorest ones give more floating, indirect answers like “studies have not shown etc.” Should you – or should you not! – that is the question!! (expert H, general comment).	• Generally, I feel it is “bad service” to write very short and then refer to many enclosed articles in an un-prioritized order. Then the enquirer has to find the answer himself, especially when the enclosed articles have different conclusions (expert F, general comment). •One response distinguishes itself from the others with inadequate background information, even though it is possible that the response is based on literature and sources that are not referred to (expert I, specific comment). • I miss a summary; this makes it difficult to get an overview of the response (expert J, specific comment). • This response does not contain anything that resembles a specific advice. The sentence “should be assessed based on a clinical risk-benefit assessment” is a triviality. Is not this always true?? (expert G, specific comment)
External experts	• The response is concise. It does not contain unnecessary details, and is presented with a clear conclusion that is logical and practical and useful (expert A, specific comment). • Well-done and thorough review of all drugs involved. A clear, sensible and practically useful conclusion too. The response witnesses considerable clinical insight. (…) Good argumentation and (…) some background information that supports the given advice (expert A, specific comment). • (…) Since the responses are supposed to be relevant for clinicians, it is important that the centres have good and transparent routines for collection of evidence, and that it is possible to view the documentation. At the same time, advice must provide the basis for clinical decision-making. (…) (expert C, general comment). • To me, it is crucial that they dare making a specific decision (expert D, general comment).	• All responses conclude that the evidence is sparse, but not all of them dare to draw a useful conclusion, and that is what you are lacking as a physician, whom is supposed to use the answer. The physician has to make a decision (expert B, general comment). • The conclusion is to “be cautious”. This is your starting point when you ask for advice (…) (expert C, specific comment). • If you enclose an article, it is important to comment WHETHER and WHAT the enquirer should read that gives him/her supplementary information to the response, otherwise you should not enclose it (expert B, specific comment). • Several kinds of advice on possible measures of action are given, but no prioritization or concrete order is suggested. That may be correct, because no “perfect” answer exist. However, I would prefer that they were more concrete, e.g. suggested one or two measures to be tried first (expert A, specific comment).

**Table 3:** Characteristics increasing and decreasing the quality of responses from drug information centres (DICs)

**Characteristics increasing the quality of responses**		
Presented data	Advice and conclusions	References/enclosed articles
*•* Correct *•* Relevant to the query *•* Clinical relevance interpreted and commented *•* All parts of a query answered *•* Synthesized (“digested”) *•* Abbreviations, definitions and concepts explained *•* States whether an updated literature search has been done *•* Logical structure	*•* Specific, clear and useful advice *•* Backgrounds/arguments for advice should be discussed *•* In accordance with the cited literature and presented data *•* If relevant, help the enquirer prioritize *•* Evidence-based *•* Problem-solving	*•* References should be presented precisely for all facts *•* Up-to-date *•* The “most important” found (e.g. new original articles) *•* For additional information *•* Enclosed articles are introduced and act as supplementary information, only – the enquirer is not required to read them *•* The content of the enclosed articles should present specific and pinpointed information
**Characteristics reducing the quality of responses**		
Presented data	Advice and conclusions	References
*•* General information not directly relevant to the query *•* Query not answered *•* Oxymoron, especially in the lack of an conclusion *•* Facts presented without interpretation of clinical significance or advice on “what to do” with the information *•* Incorrect statements or interpretation of literature	*•* Presenting advice without argumentation or scientific basement *•* “Be cautious” as the only advice *•* Risk-benefit assessment left to enquirer without further advice *•* Conclusions section lacking in longer responses	*•* Enclosed articles without any further presentation/information *•* Use of case reports and animal studies, especially without interpreting their clinical relevance

The characteristics are based on review of qualitative statements made by external (general practitioners) and internal (clinical pharmacologists) experts evaluating 42 responses to queries posed to seven Scandinavian drug information centres (DICs). Note that this is based on their individual comments, and that no consensus has been reached among the experts, concerning these characteristics

#### Presentation of background documentation and references/enclosed literature

With a few exceptions, the experts judged the pharmacological content in the responses to be concordant. Responses to the same query grossly differed in some instances in the number and types of references included. Both internal and external experts commented on this issue. For the responses to one query, an external expert stated: *The background documentation found seems to be relatively similar between centres because the same adverse effects and precautions are mentioned. However, the responses vary in terms of the use of primary and secondary articles.* Several experts introduced the terms “weak” and/or “thin” references. Although the terms were not further explained, they were used for responses referring to mainly or only, secondary and tertiary sources, e.g. the Summary of Product Characteristics (SPCs) and drug interaction databases. None of the experts explicitly stated that they preferred the use of primary references; however, one of the internal experts stated: *Secondary literature is used as references; sometimes they [*i.e. *the DICs] only refer to former responses to queries at the DIC. When is this appropriate?* None of the responses gave an overview over all articles that had been reviewed preparing the response. Thus, the experts were not aware of whether staff members preparing responses to the same query actually reviewed the same evidence, but ultimately chose to refer to different sources.

#### Provision of advice/conclusions

Both internal and external experts were concerned whether specific conclusions and/or advice were given, and seem to expect rather specific advice on how to handle the cases, especially for the patient-specific queries. There were large differences between responses in terms of whether or not advice was given and if given, how specific it was. Both external and internal experts commented on the lack of concordance. As an example, one of the external experts stated that the responses to one query *varied extensively in their ability to draw conclusions relevant for practice.* Small variations in language could result in different assessments on whether or not an advice was given. For example, a recommendation *to consider discontinuing diazepam* was assessed less precise than *we recommend discontinuing diazepam*. In one written response, the following conclusion was given: “We have not found any documentation implying that discontinuation of treatment is necessary.” In relation to this, several experts commented that the staff member preferably should have stated that the recommendation was to continue treatment.

#### The use of definitions of concepts/explanation of abbreviations

Both expert groups gave many examples of medical and pharmacological terms and/or abbreviations that they felt should be explained more thouroughly in the responses. For example, terms like *double-dummy*, *drug holiday*, *confounding by indication*, *weight-adjusted dosage*, *stereochemistry*, and abbreviations like *SNRI*, *PPHN*, *C*_*max*_, *t*_*max*_ and *AUC* were used without further explanation. One external expert stated: *I do not understand this weight-adjusted dosage. Should I do something?* and *How do you know whether the mother of the baby is a “rapid acetylator”*? Several experts further criticised the lack of translation of study findings into clinically meaningful information. One internal expert stated: *The solubility in water and ethanol is mentioned without commenting the significance of fat solubility on the degree of absorption.* The presentation of results from animal studies lacking generalizability to humans was also criticised.

### Assessment by the plain language expert

The plain language expert emphasised the importance of the responses giving competent and clear answers to the queries. The enquirer should theoretically be able to understand and use the response in an appropriate way in the treatment of a specific patient. The plain language expert did not identify features that gave her reason to believe that the responses did not fulfil these intentions. However, the expert pointed out that there was room for improvement. The expert took into account the fact that the communication took part between health care professionals. However, the expert argued that although the medical content is familiar for both parties, repeating or recapitulating information could nevertheless increase the easiness of reading the text—and thereby enhance the understanding of the response. This procedure ensures a common understanding of the content between the staff members and the readers (enquirers, or in this case, expert medical reviewers).

Table 1 shows the mean, median, minimum and maximum score of each of the eight language quality criteria, as well as arguments for including the specific criteria. The mean sum score per response was 21.1 (65.9%) out of a maximum of 32. Figure S1 depicts the distribution of sum scores for each of the responses.

## Discussion

The results from the present study indicate that the quality of structure and language of responses to drug-related queries in Scandinavian DICs is by and large satisfactory to good. Still, there is room for further improvements. The analysis of the experts’ assessments has added to our understanding of elements affecting the quality of these responses. In particular, the majority of experts emphasised the importance of giving specific, evidence-based advice, and relatively small nuances in expressions between responses may have resulted in different interpretations in terms of whether advice was specific or not. The use of phrases like “be cautious” and “perform a risk-benefit assessment” (Table 3), in the lack of more specific information on how to handle a situation, may not be useful for the clinician. Most queries posed to Scandinavian DICs are patient-specific [21], and information given in the responses needs to be applied to the particular case [2] and be operational. Scandinavian DICs include a staff of pharmacists and residents and consultants in clinical pharmacology with education and training in literature search, interpretation of published studies and experience with provision of decision support. The desire for specific advice among our external experts might be interpreted as a vote of confidence in the DICs. It is important, however, that working methods are transparent so that the enquirer may assess whether a given advice is in accordance with the existing and presented literature.

There seem to be different opinions among the experts as to whether it is sufficient to use secondary/tertiary sources and formerly answered queries to produce responses, as opposed to primary articles; however, this issue needs to be further investigated. The use of appropriate and credible sources along with critical literature evaluation skills [2] may be more important than exactly what type of source is used. The great workload of some centres may limit their ability to scrutinise the primary literature, as this kind of searching is time consuming [21, 22]. Especially for frequently asked queries where the evidence is comprehensive, e.g. use of antidepressants during pregnancy and lactation, the use of secondary and tertiary sources may be preferable from the staff members’ point of view. However, to ensure the responses being up-to-date, it is important to search for recently published primary or review articles.

Both expert groups commented on the lack of explanation of medical terms and abbreviations. Several external experts specifically mentioned medical terms (e.g. weight-adjusted dosage) that they did not understand the meaning of, and therefore did not know how to handle. A potential problem sending written responses to enquirers without verbal communication is the lack of assurance that the information and/or advice are interpreted as intended. Readers are individuals [23], and texts are read in another context than they are written [19]. Several experts criticised the lack of translation of study findings into clinically meaningful information. Rather than just cite sources passively, they recommended commenting and explaining data and results with regard to individual patient treatment and clinical practice. This suggests that it is important for DICs to maintain and develop this type of competence among the staff.

In addition to the explanation of medical and pharmacological terms and abbreviations, the plain language expert presented and assessed several factors that may contribute to better readability of the responses, all based upon plain language theory (Table 1) [10, 14]. The plain language method implies adjusting written information to the recipients’ need, structure the document clearly, use informative headings, apply active voice and explain difficult, but necessary words. The aim is to ensure that the most important informational content is identified and understood by the reader as intended by the author of the document [14]. Interestingly, responses that did well in the medical experts’ analyses did not necessarily score high in the language assessment and vice versa. Especially, our medical experts did not seem to pay much attention to the criteria 2, and 4 to 6 (Table 1). The lowest scores in the assessment of language quality were given for features that may relate to the DICs scientific style in writing, e.g. the use of passive voice instead of active, which is a common style for writing scientific articles. In addition, writing a scientific text often encompasses the need to reduce the number of words, thereby compressing the language. Whether writing more plain language actually is necessary for enquirers and staff members to understand each other is unknown. Although not all criteria may be useful in relation to the DICs’ responses, the present study underlines the importance of precise formulations, e.g. in relation to giving advice. It may not be advisable to leave to the enquirer to understand and interpret what is more or less implicitly stated in a text.

Although the number of previous studies within this field is scarce, several of the issues pointed out in this article have been mentioned by the UK Medicines Information Centres in their “Guide to writing medicines Q & As” [24] and checklists for quality assurance of queries and answers [25] available online. Included in the checklist is that the information given should be concise and presented in a logical order, no unreferenced key statements should exist, the summary should reflect key points accurately and completely, no mistakes should be made in the referencing, and a primary literature search (including the use of Embase/Medline) should always be performed [25].

### Strengths and limitations

No studies have previously published results from quality assessments of written DIC responses using qualitative data and assessment by a plain language expert. Although identical queries were posed to the centres, we did not expect seven identical responses to each of these. Each centre has its “style” and working methods (Table S1), and this clearly affected the responses. In addition, the choice of literature to refer to, interpretation of documentation and the exact structure and wording of the response are examples of working procedures that cannot be 100% similar between individual staff members. The lack of concordance between DIC responses in terms of advice illustrates the difficulties in streamlining the preparation of these responses. However, this lack of concordance was also very useful, as we thereby could identify a potentially important quality criterion in DICs’ responses; to give specific, clinically useful advice.

The method for collecting the data required that internal and external medical experts supplied written comments. As such, one might criticise the level of systematic data collection. Surely, experts contributed in a variable degree to the qualitative comments. Moreover, most comments represented the opinion of single experts and no attempt was made to reach consensus among the experts with this respect. Our external experts may not be representative for the average user of the Scandinavian DICs, as they were familiar with and perhaps had an inherent positive attitude to the DICs. In addition, the monetary incentive may have introduced a bias, as the external experts may have felt obligated to judge the responses better than they otherwise would. However, the assessment of responses was quite time consuming, and we feared we would not be able to recruit GPs for this task without any financial reward.

The wording of the only query in the assessment form specifically formulated for qualitative information may have increased the experts’ focus on advice and conclusions, and introduced a bias. The experts reviewed responses to prefabricated study queries rather than self-provided queries, and the comparison of several responses to the same query might have made them especially strict in their assessments. However, it may also have helped them realise what the essential factors in the “best responses” actually were, which was our intention.

We focused on written responses only, because all the included DICs produce written responses to most queries. However, some of the centres give oral responses by telephone instead and/or in addition. Quality-assuring verbal responses would require tape-recording of telephone calls, with an increasing possibility of violation of the blinding. In this study, we have no confirmation that unblinding took place, but some DICs and staff members did express suspiciousness towards some queries. This may have caused them to respond more thoroughly to these queries than they otherwise would have done.

We posed only six queries to the DICs during this study. Although these were typical for the centres included, mainly patient-specific, the responses may not be representative for all the responses given. Nevertheless, the importance of this study does not necessarily lie in the quality of each response, but in the possibility to compare different responses to the same query against each other.

Our plain language expert usually works with texts intended for lay people and had to do some adjustments when working with DIC responses. The responses are specific answers to unique, mostly patient-related queries, rather than general information. Therefore, the expert did not assess the necessity of an introduction, background information and referral to further information, as normally would have been done. The Norwegian plain language expert had experience in evaluation of both Swedish and Danish language. The principles of plain language are generally the same for all three languages, but being Norwegian, we cannot rule out that the expert may have been biased in the evaluation of texts in the three different languages. Due to the cost of the language evaluation, we limited the assessment to one expert only and the scores of the language criteria are based on qualitative assessments. Applying an assessment of the language in these responses have extended and improved our understanding of the importance of structure and language style in these responses, although we may not feel that all criteria, e.g. the use of active voice, were suitable for our context.

## Conclusion

The quality of structure and language of responses to drug-related queries posed to seven Scandinavian DICs was generally satisfactory to good. Improvements suggested by medical and language experts include providing specific advice on action, and presenting and defining professional terms and abbreviations in order to increase both the readability and the understanding of the responses.

## Electronic supplementary material

Table S1 (DOCX 14 kb)

Table S2 (DOCX 16 kb)

Figure S1 Distribution of sum scores of language quality of responses produced by Scandinavian drug information centres (DICs) to six fictitious queries (six queries were posed to seven different DICs, giving a total of 42 responses). The sum score is based on eight quality criteria developed and assessed by a plain language expert with a Master of Arts in Rhetoric. Each criterion was scored from 0 (poorest quality) to 4 (highest quality). Thus, minimum possible sum score was 0, and maximum was 32. (DOCX 26 kb)
