# Supplementary material for: Quality assessment of structure and language elements of written responses given by seven Scandinavian drug information centres
Source: Eur J Clin Pharmacol. 2017 Feb 5;73(5):623–31. doi: 10.1007/s00228-017-2209-3 (PMC5384946; doi:10.1007/s00228-017-2209-3)
Supplement: Supplementary file 1 — (DOCX 14 kb) [file 228_2017_2209_MOESM1_ESM.docx]

# Supplementary material

**Table 1:** Key information on participating Scandinavian Drug Information Centres (DICs) and their routines for preparing and quality assuring responses.
Six fictitious queries were sent to each of the centres.

| ***Centre*** | **Country** | **Occupation of staff members responding to the six queries** | **Written routines for preparing responses^a^** | **Use of checklists for which sources to search^a^** | **Use of countersignature^a^** | **Responses discussed in meetings^a^** |
| --- | --- | --- | --- | --- | --- | --- |
| *1* | Denmark | Pharmacist: 1 Residents: 5 | Always | Always | Always | Always |
| *2* | Norway | Pharmacist: 5 Resident: 1 | Always | Sometimes | Often | Sometimes |
| *3* | Norway | Pharmacist: 3 Resident: 3 | Seldom | Seldom | Often | Sometimes |
| *4* | Norway | Pharmacist: 6 | Seldom | Sometimes | Always | Often |
| *5* | Norway | Pharmacist: 5 Resident: 1 | Always | Often | Often | Sometimes |
| *6* | Denmark | Residents: 6 | Often | Always | Always | Always |
| *7* | Sweden | Pharmacist: 1 Residents: 2 Clinical pharmacologist: 3 | Often | Often | Often | Often |

^a^ These categories are answers to the query *How often are these systems of quality assurance used in the daily work, responding to queries?*
The information is based on data provided by the leaders of the DICs immediately before the study was undertaken.
