# Supplementary material for: Quality assessment of structure and language elements of written responses given by seven Scandinavian drug information centres
Source: Eur J Clin Pharmacol. 2017 Feb 5;73(5):623–31. doi: 10.1007/s00228-017-2209-3 (PMC5384946; doi:10.1007/s00228-017-2209-3)
Supplement: Supplementary file 2 — (DOCX 16 kb) [file 228_2017_2209_MOESM2_ESM.docx]

**Table 2:** The six drug-related queries from general practitioners (GPs) were simultaneously submitted to seven Scandinavian drug information centres (DICs) during a study period of eight weeks. All GPs asked each query on the same day. Queries were originally asked in Norwegian, Danish and Swedish languages. The table has previously been published in another article from our group (20).

| **Query no.** | **Query** |
| --- | --- |
| I | A female patient presents with deep, infected pockets close to the jaw bone, and needs to have these rinsed every 4^th^ to 5^th^ week for the next six months. The patient uses alendronate 70 mg once weekly. Should alendronate be discontinued during treatment? |
| II | A male patient with formerly performed gastric bypass needs treatment for *Helicobacter pylori* infection due to symptomatic ulcus. The GP wants to start treatment with pantoprazole 40 mg once daily, metronidazole 400 mg twice daily and amoxicillin 750 mg twice daily. Will the absorption of the drugs be reduced, and are dosage adjustments necessary? |
| III | A pregnant women manifests with moderate depression (MADRS^1^ score 29), and there is indication for treatment with an antidepressant. What antidepressant is the first choice of drug during pregnancy? |
| IV | A GP has registered an increasing use of Ginkgo biloba in nursing care homes and home nursing services. He (she) has also registered that ginkgo might increase bleeding time. What documentation exists on this topic, and what is the relevance for concomitant use of e.g. warfarin, acetylsalicylic acid, clopidogrel and enoxaparin? |
| V | A male patient, 75 years old, has gradually developed impaired cognition for the last 5-6 months (MMS^2^ score 18 at examination). He has essential hypertension treated with atenolol 100 mg once daily and losartan/hydrochlorothiazide 100/12.5 mg once daily. His blood pressure was 130/90 mmHg at the latest appointment. He also uses simvastatin 40 mg and acetylsalisylic acid 160 mg once daily. He uses paroxetine 40 mg in the morning for anxiety/depression, diazepam 5 mg as needed and promethazine/propiomazine^3^ 50 mg at night for sleep. He also uses tolterodine 2.8/4 mg^4^ once daily for overactive bladder (dosage increased from 1.4/2 mg^4^ three months ago). The patient does not smoke. Can any of these drugs, or drug interactions, increase the risk of impaired cognition? |
| VI | A female patient, 13 weeks post partum, presents with active ulcerative colitis. She has earlier been treated with sulfasalazine 500 mg x 3 (discontinued during pregnancy). Can she use sulfasalazine while breast-feeding? |

^1^ MADRS, Montgomery and Asberg depression rating scale. ^2^ MMS, Mini Mental Status. ^3^ Promethazine is marketed in Norway and Denmark, but not in Sweden. For the Swedish query, we used the drug propiomazine. Both drugs are derivates of phentiazines with antihistaminic and anticholinergic effects. ^4^ Tolterodine is marketed as 2 and 4 mg depot capsules in Norway and Sweden, and as 1.4 and 2.8 mg depot capsules in Denmark.
