# Supplementary material for: Quality assessment of structure and language elements of written responses given by seven Scandinavian drug information centres
Source: Eur J Clin Pharmacol. 2017 Feb 5;73(5):623–31. doi: 10.1007/s00228-017-2209-3 (PMC5384946; doi:10.1007/s00228-017-2209-3)
Supplement: Supplementary file 3 — Distribution of sum scores of language quality of responses produced by Scandinavian drug information centres (DICs) to six fictitious queries (six queries were posed to seven different DICs, giving a total of 42 responses). The sum score is based on eight quality criteria developed and assessed by a plain language expert with a Master of Arts in Rhetoric. Each criterion was scored from 0 (poorest quality) to 4 (highest quality). Thus, minimum possible sum score was 0, and maximum was 32. (DOCX 26 kb) [file 228_2017_2209_MOESM3_ESM.docx]

**Figure 1**
